# Breed and timepoint-based analysis of chicken harderian gland transcriptome during Newcastle disease virus challenge

**DOI:** 10.3389/fmolb.2024.1365888

**Published:** 2024-06-10

**Authors:** Venkata Krishna Vanamamalai, E. Priyanka, T. R. Kannaki, Shailesh Sharma

**Affiliations:** ^1^ National Institute of Animal Biotechnology (NIAB), Hyderabad, Telangana, India; ^2^ Regional Centre for Biotechnology, Faridabad, Haryana, India; ^3^ ICAR-Directorate of Poultry Research, Hyderabad, Telangana, India

**Keywords:** Newcastle disease, harderian gland, leghorn and fayoumi, differential resistance patterns, long non-coding RNAs, gene-lncRNA Co-expression analysis, qPCR validation

## Abstract

**Introduction:** Newcastle disease is a highly infectious disease caused by the Newcastle Disease Virus (NDV) and has a devastating financial impact on the global chicken industry. It was previously established that Leghorn and Fayoumi breeds of chicken exhibit variable resistance against NDV infection. The harderian gland is the less studied tissue of the chicken, known to play an essential role in the immune response.

**Methods:** Our previous study, we reported differential gene expression and long noncoding RNAs (lncRNAs) between challenged and non-challenged chickens in the Harderian gland transcriptomic data. Now, we report the analysis of the same data studying the differential expression patterns between Leghorn and Fayoumi and between different timepoints during disease. First, the pipeline FHSpipe was used for identification of lncRNAs, followed by differential expression analysis by edgeR (GLM), functional annotation by OmicsBox, co-expression analysis using WGCNA and finally validation of selected lncRNAs and co-expressing genes using qRT-PCR.

**Results:** Here, we observed that Leghorn showed a higher number of upregulated immune-related genes than Fayoumi in timepoint-based analysis, especially during the initial stages. Surprisingly, Fayoumi, being comparatively resistant, showed little difference between challenged and non-challenged conditions and different time points of the challenge. The breed-based analysis, which compared Leghorn with Fayoumi in both challenged and non-challenged conditions separately, identified several immune-related genes and positive co-expressing cis lncRNAs to be upregulated in Fayoumi when compared to Leghorn in both challenged and non-challenged conditions.

**Discussion:** The current study shows that Leghorn, being comparatively more susceptible to NDV than Fayoumi, showed several immune-related genes and positive co-expressing cis lncRNAs upregulated in challenged Leghorn when compared to non-challenged Leghorn and also in different timepoints during challenge. While, breed-based analysis showed that there were more upregulated immune genes and positive cis-lncRNAs in Fayoumi than Leghorn. This result clearly shows that the differences in the expression of genes annotated with immune-related GO terms and pathways, i.e., immune-related genes and the co-expressing cis-lncRNAs between Leghorn and Fayoumi, and their role in the presence of differences in the resistance of Leghorn and Fayoumi chicken against NDV.

**Conclusion:** These immune-genes and cis-lncRNAs could play a role in Fayoumi being comparatively more resistant to NDV than Leghorn. Our study elucidated the importance of lncRNAs during the host defense against NDV infection, paving the way for future research on the mechanisms governing the genetic improvement of chicken breeds.

## Introduction

Newcastle disease is a highly infectious disease that has a devastating financial impact on the global chicken industry. It is caused by the Newcastle Disease Virus (NDV) or Avian Ortho avulavirus-1, which belongs to Kingdom Orthornavirae, Family Paramyxoviridae and Genus Avian Orthoavulavirus (El-Hamid et al., 2020). While vaccinations are widely available, Newcastle Disease is known to cause fatal effects in poultry. Although the immune response due to vaccination is vital, it is essential to study the differences in the resistance to Newcastle Disease due to the genetic variations among poultry breeds (Kapczynski et al., 2013).

In the previous study by M.S. Tarabany (Mahmoud S, 2019), the authors evaluated the immune response against NDV live attenuated and inactivated vaccines in purebred Leghorn, Fayoumi, male Fayoumi × female Leghorn (FL crossbred) and male Leghorn × female Fayoumi (LF reciprocal crossbred) Chicken. This study concludes that the purebred Fayoumi showed the highest antibody titer and lowest mortality rate, followed by the crossbreeds. In contrast, purebred Leghorn showed the lowest antibody titer and highest mortality rate. This differential immune response in the different breeds of *Gallus* (Chicken) laid the foundation for further research where the genetic differences were studied.

The harderian gland is the less studied tissue of the chicken, present behind the eye. Although its functions have not been studied well, it is known to play an essential role in immune response. The comparative analysis of the harderian gland to other crucial immune system organs showed that the harderian gland plays a key role in immune response (Vanamamalai et al., 2021). A study on the harderian gland of Leghorn and Fayoumi breed Chicken during Newcastle Disease showed the role of genes differentially expressing between the two breeds. This study shows that the Fayoumi breed, being comparatively more resistant to NDV than Leghorn, expressed more immune system-related genes than Leghorn during normal and Newcastle Disease Virus challenge conditions (Deist et al., 2018).

Long noncoding RNAs (lncRNAs) are transcripts with a length greater than 200 nucleotides and do not code for a functional protein. They are synthesised by RNA Polymerase - II and possess a 5′cap. Several lncRNAs, known as poly-adenylated lncRNAs, possess a 3′poly-A tail, while some non-polyadenylated lncRNAs are known to contain a triple helix (Wilusz et al., 2012). Although there is a debate regarding the functionality of lncRNAs, the growing number of lncRNA research shows the importance of lncRNAs. They can fold into complex structures, which enables them to interact with different molecules like DNA, other types of RNAs and even specific proteins (Anna et al., 2018). The primary function of lncRNAs is gene regulation at multiple levels, including DNA, RNA, and protein. The lncRNAs are involved in various biological activities, including gene activation and inactivation by modulating the chromatin, interacting with DNA and histone proteins, modulation of the transcriptional activity by interacting with mRNAs and transcriptional machinery including transcription factors, post-transcriptional regulation by sequestering the proteins to form lncRNA-protein complexes which modulate splicing and turnover of mRNAs and translational regulation by modulating translation assembly and binding directly with other types of RNAs. The lncRNAs are also involved in regulating miRNAs by blocking the miRNAs through miRNA sponging. The lncRNAs can also regulate proteins by binding through RNA-protein interactions. This gene regulation of lncRNAs, in turn, regulates various physiological activities of cells, including cell differentiation, cell growth and responses to various stimuli. The lncRNAs also play vital roles in muscular, nervous, cardiovascular and immune systems (Statello et al., 2021). In immune system-related processes, lncRNAs play significant roles in haematopoietic development, myeloid differentiation, CD4^+^ t-cell differentiation, inflammation activation and restriction through lncRNA-DNA, lncRNA-RNA and lncRNA-protein interactions (Chen et al., 2017).

In our recent study on trachea transcriptome (Vanamamalai et al., 2023), we have identified lncRNAs and performed differential expression analysis in three different types–challenge-based analysis, i.e., between non-challenged and challenged samples, breed-based analysis, i.e., between Leghorn and Fayoumi, and timepoint based analysis, i.e., between different timepoints–2, 6, 10 days post-challenge (DPC). However, in our previous study on the transcriptomic data of the harderian gland (Vanamamalai et al., 2021), we have identified the genes and long noncoding RNAs differentially expressing between normal samples and NDV-challenged samples of Leghorn and Fayoumi breeds separately, i.e., challenge-based analysis only, which made us understand the role of genes and lncRNAs during the infection stages in both breeds. In continuation to that, in this work, we analysed the same data to identify the genes and lncRNAs that were differentially expressed between Leghorn and Fayoumi samples at both normal and NDV-challenged conditions at three different timepoints, i.e., Breed based analysis and between different timepoints, when challenged with NDV, i.e., timepoint based analysis. We predict that this helps us to understand the role of genes and long noncoding RNAs, which could be involved in the Fayoumi breed becoming comparatively more resistant to NDV than the Leghorn breed chicken.

## Methods

### Transcriptome sequencing data

The transcriptomic data of the harderian gland of Leghorn and Fayoumi chicken was downloaded from the publicly available database EBI-ENA, with project ID PRJEB22672 (Deist et al., 2018). The dataset comprises 94 samples: 46 normal samples and 48 NDV-challenged samples. The details of the sample dataset are shown in Supplementary Table S1. The computational pipeline FHS pipe, as explained in our previous work (Vanamamalai et al., 2023).

### Identification of long noncoding RNAs using FHSpipe

The pipeline includes various steps – Quality filtering using Fastp (Shifu et al., 2018), mapping using HISAT2 v2.2.1 tool (Kim et al., 2019) and the latest reference genome of Chicken (GRCg7b), assembly using Stringtie v2.1.4 tool (Pertea et al., 2015). The assembled transcripts were then annotated with different class codes using the tool GFFCompare v0.11.2 (Pertea and Pertea, 2020). The sequences of class codes “I,” “U,” and “X” were selected and extracted using the Bedtools v2.28.0 (Quinlan and Hall, 2010). The extracted sequences were subjected to various filters–length filter (minimum 200 nucleotides), ORF filter (maximum 300 nucleotides/100 amino acids), Pfam filter (no hits) using RPSBlast v2.11.0+ (Christiam et al., 2009) against the Pfam database (Mistry et al., 2021) with an e-value 1e-3 and coding potential filter using Coding potential calculator 2 (CPC2) (Kang et al., 2017). The sequences with noncoding tags were filtered and subjected to BlastN v2.11.0+ (Christiam et al., 2009) with 100% query coverage and 100% identity against other noncoding RNA databases like eukaryotic transfer RNA database (Chan and Lowe, 2009), Silva ribosomal RNA database (Christian et al., 2013) and miRbase (Kozomara et al., 2019) as final potential long noncoding RNAs. These are searched against NONCODE database version 6 (Zhao et al., 2021) using BlastN (Christiam et al., 2009) with 100% query coverage and 100% identity to identify the known and novel lncRNAs. The identified lncRNAs were plotted across the Chromosomes according to their locus positions using Phenogram (Wolfe et al., 2013). Previously (Vanamamalai et al., 2021), the lncRNAs were extracted separately at each timepoint, which generated many duplicates between each timepoint. So, now lncRNAs are identified from the entire dataset at once, which gives a non-redundant list of lncRNAs.

### Differential expression analysis of genes

FHSpipe generates read counts for the genes and the long noncoding RNAs, which were further used to perform differential expression analysis using edgeR v3.34.0 with Generalised linear model (GLM) (Robinson et al., 2010). Contrasts were written to identify the genes differentially expressed between Leghorn and Fayoumi at both conditions (Breed-based analysis) at three timepoints and between different timepoints of challenged samples (Timepoint-based analysis). The genes and lncRNAs with an FDR value below 0.05 were filtered as significant differentially expressed genes (DEGs) and significant differentially expressed lncRNAs (DElncRNAs). Using the tool Circos v0.69.8 (Krzywinski et al., 2009), the chromosomal localisation of differentially expressed genes and differentially expressed lncRNAs was plotted.

### Gene ontology, pathway analysis and GSEA

As described earlier (Vanamamalai et al., 2023), GO functional annotation of the identified DEGs was performed using a standalone tool, OmicsBox v2.1.14 (BioBam Bioinformatics, 2019), which includes several steps: BLAST (Altschul et al., 1990), Interpro scan (Philip et al., 2014), Gene ontology mapping and annotation (Gotz et al., 2008), eggNOG mapping (Huerta-Cepas et al., 2019), and combined pathway analysis against Reactome (Fabregat et al., 2018) and KEGG (Kanehisa and Goto, 2000) databases.

GO functional enrichment analysis was performed separately of all the conditions using the GSEA (Gene Set Enrichment Analysis) module in OmicsBox (Subramanian et al., 2005) to detect the significantly different Gene Ontologies and pathways in challenge-based, breed-based and timepoint-based analysis. Various options, including the number of gene set permutations, weighted enrichment statistic and cutoffs (*p* < 0.05, FDR<0.25, |NES|>1), were used as mentioned previously (Vanamamalai et al., 2023).

### Co-expression analysis

WGCNA (Weighted Gene Correlation Network Analysis) v1.70.3 (Langfelder and Horvath, 2008) was used to perform the co-expression analysis of DElncRNAs with the DEGs, which is further used for functional annotation of the DElncRNAs. Co-expression analysis was conducted individually for each condition for all the DEGs and DElncRNAs. The interactions between DEGs and DElncRNAs, involved in the Immune system processes and with a minimum weight cutoff of 0.01, were chosen and visualised using Cytoscape v3.9.1 (Shannon et al., 2003) in the form of networks.

### Cis–trans and functional analysis of lncRNAs

Based on chromosomal localisation, the DEGs and DElncRNAs co-expression interactions can be cis and trans interactions. The co-expressing DEG and DElncRNA were found on the same chromosome in cis, whereas they were found on separate chromosomes in trans interactions (Guttman and Rinn, 2012; Zhao et al., 2020). The type of interaction between the DEGs and the co-expressing DElncRNAs was determined through their chromosomal positions. The functions of DElncRNAs were predicted using the functions of the DEGs co-expressing with these DElncRNAs. The pathways for each gene obtained from Gene-ontology analysis were taken and assigned to the respective co-expressing lncRNA.

### Gene–transcription factor interaction analysis

The gene-transcription factor interaction was determined using the standalone tool MEME-suite v5.3.2 (Bailey et al., 2009). As mentioned in our previous studies (Vanamamalai et al., 2021; Vanamamalai et al., 2023), the 5′UTR of 5 KB size of all the DEGs were extracted and analysed for the motifs using the MEME tool under MEME-suite. Using the Tomtom tool of MEME-suite, the obtained motifs were then compared to the JASPER2022 Vertebrates database to identify the potential transcription factors binding to these motifs (genes). These were scanned against the Animal transcription factor database v4.0 (Shen et al., 2023) to obtain the transcription factors of chicken. This data was used to plot a gene-TF network using Cytoscape v3.9.1 (Shannon et al., 2003).

### Gene–miRNA interaction analysis

The microRNAs interacting with DEGs were obtained using miRNet v2.0 (Chang et al., 2020), an online platform for miRNA target prediction. This tool uses the miRNA target gene data collected from well-annotated databases - miRTarBase v8.0, TarBase v8.0 and miRecords. This tool takes a list of either official gene symbols Entrez IDs or Ensembl IDs as input to predict gene-miRNA interaction. The output was in the CSV file format, which contains the details of miRNA ID and accession, target gene symbol and ID, Predicted miRanda Score, Literature ID and tissue type. This data is used to plot a gene-miRNA network using Cytoscape v3.9.1 (Shannon et al., 2003).

### QTL analysis

The coordinates of each QTL (Quantitative Trait Locus) were downloaded from the Animal QTL Database (Zhi-Liang et al., 2019), DEGs and DElncRNAs were collected, and QTLs were assigned to each of the DEGs using an in-home built Python script based on the positions of the QTL and DEGs.

### Validation studies

A total of 7 lncRNAs, 5 lncRNAs with co-expressing genes and 2 lncRNAs without gene, were randomly selected for validation by RT-PCR. Three pairs of co-expressing lncRNA-genes, namely, TCONS_00565961 (Lnc 1)–PIGR (Gene 1), TCONS_00210774 (Lnc 2)–TF (Gene 2) and TCONS_00128750 (Lnc 3)–DOCK8 (Gene 3) were selected from 2 vs. 6-day data. Two pairs and one lncRNA without a co-expressing gene, namely, TCONS_00176905 (Lnc 4)–BST1 (Gene 4), TCONS_00246845 (Lnc 5)–JCHAIN (Gene 5) and TCONS_00566193 (Lnc 6) were selected from 2 vs. 10-day data. One lncRNA TCONS_00279966 (Lnc 7) without a co-expressing gene was selected from 6 vs. 10-day data. Of these genes, gene JCHAIN was annotated with GO Immune system process (GO:0002376), the genes PIGR and LOC107051274 were annotated with Immune system (Reactome) category pathways and the genes BST1, TF were annotated with both immune GO and pathways. While, the gene DOCK8 was annotated with Signal transduction pathways.

The NCBI primer design tool (Ye et al., 2012) was used to design the primers to be used in the RT-PCR and the information was mentioned in Supplementary Table S2. The experimental design and methodology for the lentogenic NDV challenge were used as previously described in detail in our previous work on trachea transcriptome analysis (Vanamamalai et al., 2023). This work was performed adhering to the ethical guidelines approved by the Institute Animal Ethics Committee (IAEC/DPR/20/2). Briefly, chickens aged 21 days were grouped into two groups of 30 each–one group was inoculated with 200 μL of EID 50 ≥ 10^6^ per dose of NDV of LaSota strain, with 50 μL inoculated into each eye and nostril. Similarly, the other group was inoculated with 200 μL of phosphate-buffered saline (PBS). Hemagglutination inhibition (HI) and indirect ELISA determined the ND antibody levels as described earlier (Vanamamalai et al., 2023). Harderian gland tissue was collected from both the groups on 2, 6 and 10 DPC. Total RNA was isolated from the tissue using the Viral RNA purification kit (Himedia Pvt. LTD.) and reverse transcribed to cDNA using the High-capacity cDNA Reverse transcription Kit (Applied Biosystems, United States). Insta Q96™ real-time PCR machine (Himedia, India) was used to perform real-time PCR using Maxima SYBR Green/ROX qPCR Master Mix (2X) (MBI Fermentas, United States). The 2^−ΔΔCt^ method was used to calculate relative expression between respective timepoints with housekeeping gene β actin as the internal control. GraphPad Prism (GraphPad Software, 2024) was used to visualise the results as bar plots, including the standard error of the mean (SEM) and the significance values obtained using multiple unpaired t-tests.

## Results

### Identification of long noncoding RNAs–FHSpipe

In quality filtering, an average of about 95.67% of the reads had passed the quality filter in the 94 samples, ranging from a minimum of 73.05% to a maximum of 98.63% in different samples. The average Q30 base content was found to be 95.25% across all the samples, and the average GC content was found to be 47.49%. The mapping percentage of all the samples was in the range of 71.05%–96.28%. The average mapping percentage was 90.58%, with a median quality of 92.47%. The number of assembled transcripts in the first run of the assembly was in the range of 38,547–49,406 transcripts. After reassembly using merged GTF, the number of transcripts was found to be 28,788 in all the samples. The quality and assembly details of each sample are mentioned in Supplementary Table S3.

**FIGURE 1:**
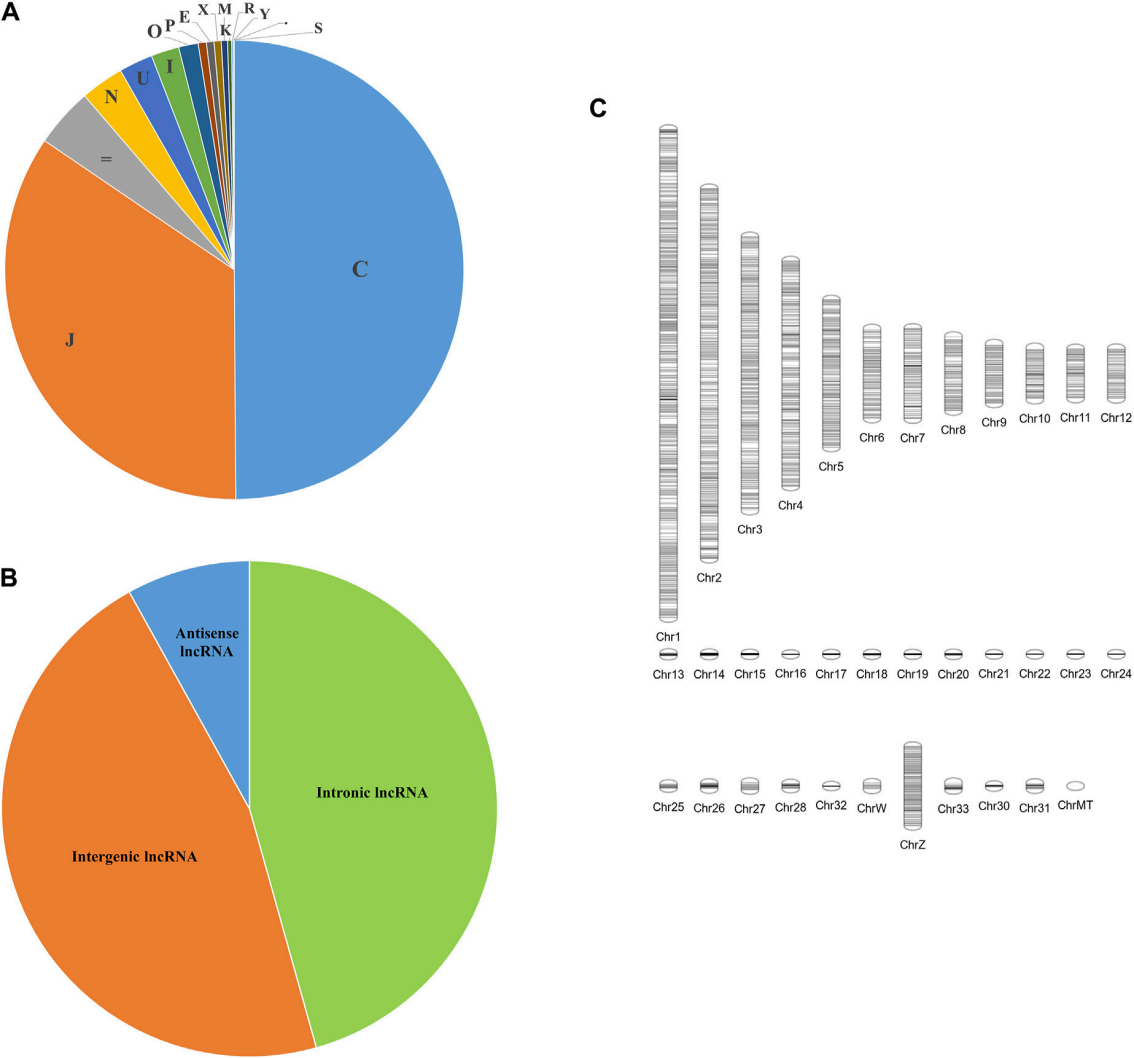
Figure showing the **(A)** Pie chart of the classification of all the transcripts into 16 class codes, **(B)** Pie chart of the classification of the identified lncRNAs into 3 different types—Intronic, Intergenic, and Anti-sense, **(C)** Chromosomal positions of all the identified long noncoding RNAs.

A total of 16 different class codes were identified, of which 34,309 sequences of class codes I, U and X were extracted initially. Figure 1A shows the pie chart showing the proportions of the 16 class codes. After processing the data with length, ORF, PFAM and coding potential filters, 29,047 sequences were obtained. Out of the 29,047 final lncRNAs, about 13,261 sequences belong to class code I, 13,445 to class code U, and 2,341 to class code X, as shown in Figure 1B. The chromosomal localisation of the identified lncRNAs plotted using the tool Phenogram were shown in Figure 1C. With default blast parameters and no identity and coverage filter, about 11 sequences showed hits against the transfer RNA database of chicken and no hits against ribosomal RNA and miRbase databases. While using 100% identity and coverage filters, no sequences were similar to transfer RNA, ribosomal RNA and miRbase databases. The BlastN against NONCODE database version 6 showed that about 23,240 sequences showed no hits and 5,807 sequences showed hits, of which 160 sequences showed hits with 100% query coverage and 100% identity. These details are shown in Table 1 Detailed information on all the identified long noncoding RNAs is mentioned in Supplementary Table S4.

**TABLE 1:** Table showing the number of transcripts discarded in different steps in the lncRNAs identification pipeline.

Step	Number of sequences eliminated	Number of sequences retained
Total	—	34,309
Length filter (<200)	0	34,309
ORF filter (>300)	2032	32,277
Pfam filter	1,116	31,161
CPC2	2,114	29,047
Final	5,262	29,047

### Differential expression analysis

**FIGURE 2:**
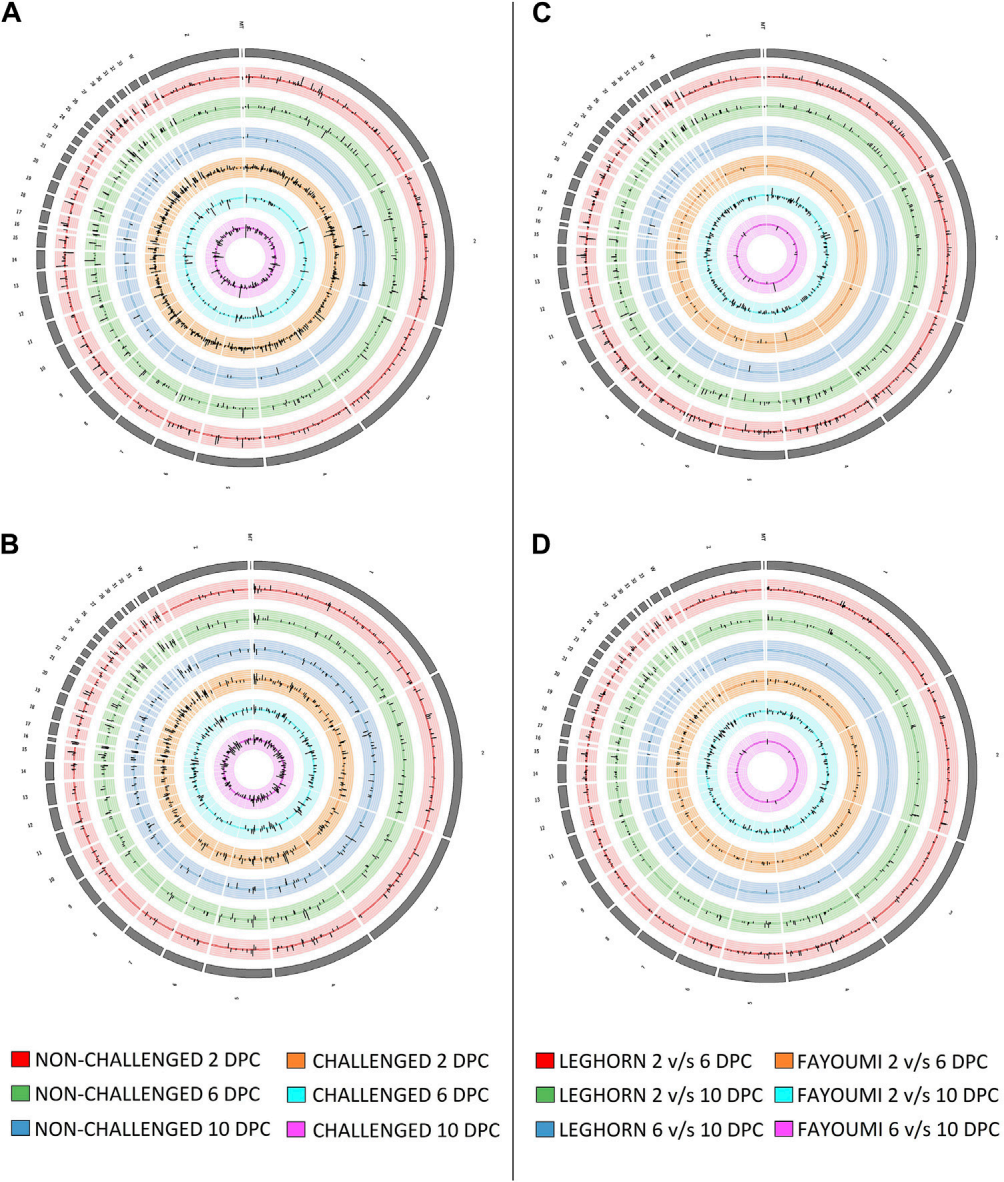
Figure showing the synteny plot of the chromosomal localisation of Differentially expressed genes **(A, C)** and differentially expressed lncRNAs **(B, D)** obtained in breed-based analysis **(A, B)** and timepoint-based analysis **(C, D)**.

Several DEGs and DElncRNAs were obtained at each timepoint, including upregulated and downregulated sequences, as shown in Table 2A for breed-based analysis and Table 2B for timepoint-based analysis. Supplementary Figure S1 shows the heatmap of the read counts of the DEGs, and Supplementary Figure S2 shows that of the DElncRNAs. Figure 2 shows the chromosomal localisation of the DEGs (A, C) and DElncRNAs (B, D) obtained in breed-based analysis (A, B) and timepoint-based analysis (C, D). More DEGs and DElncRNAs were identified in breed-based analysis than in timepoint-based analysis. The highest number of DEGs and DElncRNAs were identified in 2DPC in challenged, followed by 2DPC non-challenged. These results show that both breeds respond differently right from the initial stage of infection. In timepoint-based analysis, Leghorn showed the highest number of DEGs and DElncRNAs between 2 DPC and 6 DPC compared to Fayoumi. Although a similar number of DEGs and DElncRNAs were identified between 2 DPC and 10 DPC.

**TABLE 2:** Table showing the number of differentially expressed genes and lncRNAs at each condition. A: Breed-based analysis. B: Timepoint-based analysis.

A	Non-challenged			Challenged		
	2-day	6-day	10-day	2-day	6-day	10-day
DEGs	412	289	43	1,039	110	163
DelncRNAs	744	638	422	1,283	649	508

B	Leghorn			Fayoumi		
	2v6	2v10	6v10	2v6	2v10	6v10
DEGs	45	285	17	587	293	10
DelncRNAs	204	312	17	606	297	21

### Gene ontology analysis

**FIGURE 3:**
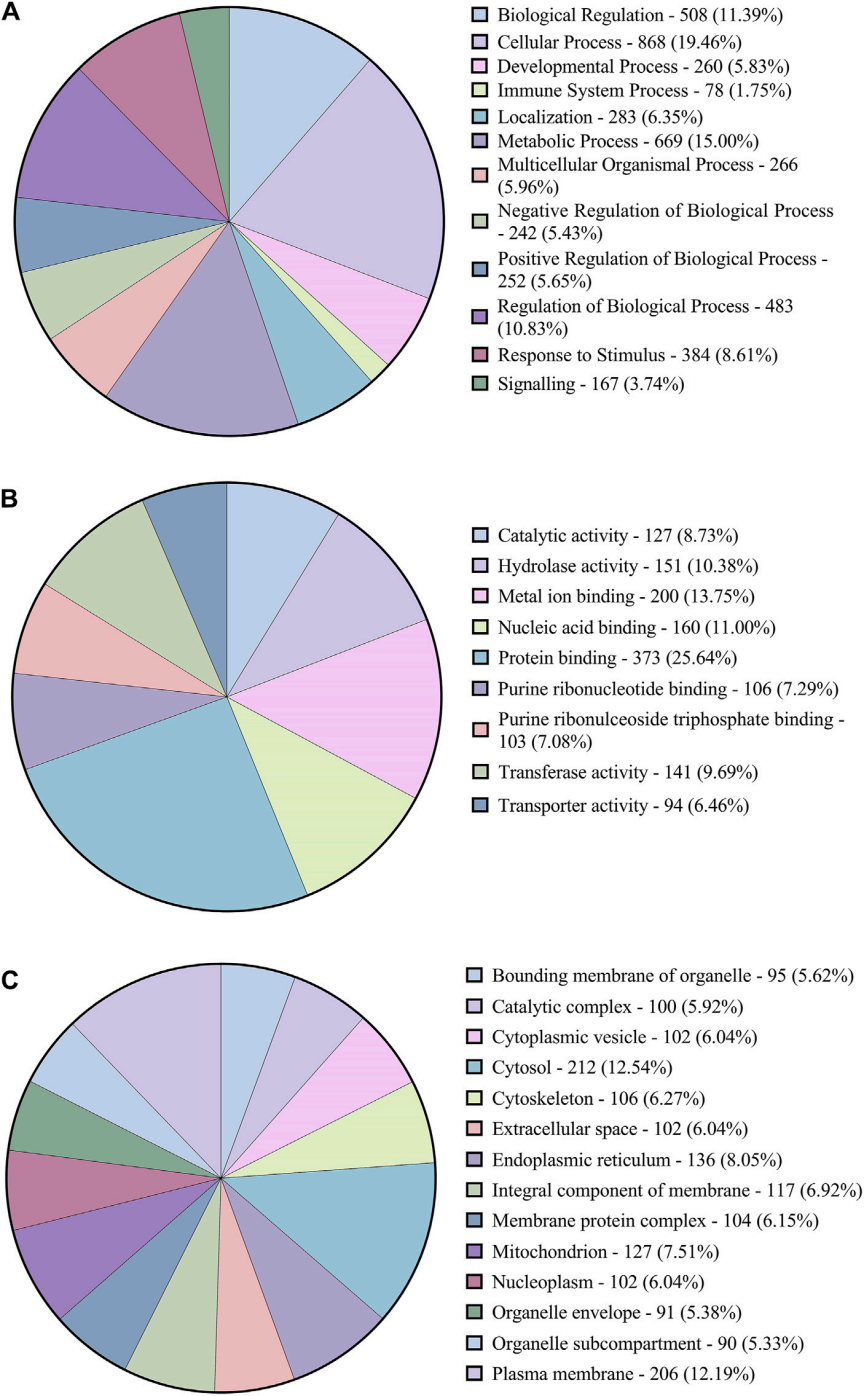
Figure showing the pie charts representing **(A)** the distribution of Level-2 Biological process GOs, **(B)** sequence distribution of Molecular function GOs and **(C)** Cellular component GOs annotated to DEGs using OmicsBox.

**FIGURE 4:**
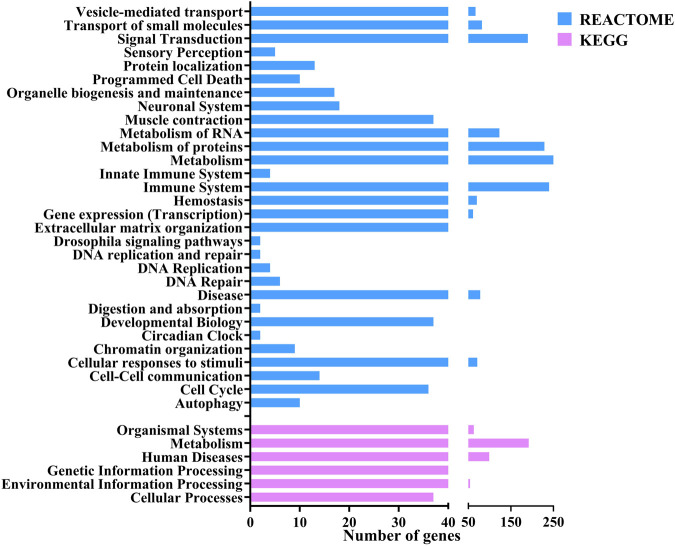
Figure showing the bar chart distribution of the categories of KEGG and Reactome pathways annotated to DEGs annotated using OmicsBox.

The functional annotation of the DEGs showed they were involved in several pathways. Figure 3 shows the Level 2 Gene ontology annotation in (A) Biological process, (B) Molecular function and (C) Cellular component plotted using GraphPad Prism (GraphPad Software, 2024). The Biological process distribution shows that 19% of the sequences were annotated with Cellular process (GO:0009987), followed by 15% with metabolic process (GO:0008152) and 2% (78) of the DEGs with Immune system process (GO:0002376). The molecular function distribution shows that 60% of the sequences were annotated with Binding (GO:0005488) and 40% with Catalytic activity (GO:0003824). The cellular component distribution shows that 74% of the sequences were annotated with Cellular anatomical entity (GO:0110165) and 26% with protein-containing complex (GO:0032991). Figure 4 shows the number of pathways per category annotated to each of the DEGs using Reactome and KEGG databases plotted using GraphPad Prism (GraphPad Software, 2024). In KEGG categories, 91 out of 247 pathways were found to be under the “Metabolism” category, followed by the “Human diseases” category with 63 pathways. The least number of pathways were found in the “Genetic information processing” category, with only 8 pathways. In the Reactome categories, 170 out of 968 pathways were found to be under the “Signal transduction” category, followed by the “Metabolism” category with 138 pathways. The least number of pathways were found in “Digestion and absorption,” with only one pathway. In addition, about 117 pathways were found in the “Immune system” category. These 117 immune system pathways were annotated to 240 DEGs. In addition, 28 DEGs were found to be annotated with both Level 2 biological process GO Immune system process (GO:0002376) and immune system (Reactome) category pathways; out of these, five were novel genes with no known official gene symbol. About 290 DEGs were found to be annotated with either immune-related GO terms or immune system pathways. The details, including Gene Ontologies (BP, MF, CC), EGGNOG annotation, pathway name, pathway category, and expression values (log2 fold change) of the DEGs identified in breed-based analysis and timepoint-based analysis, were mentioned in Supplementary Table S5.

### GO functional enrichment analysis

Several enriched/over-represented Gene ontologies and pathways were identified using functional enrichment analysis after filtering the data with *p* < 0.05, FDR<0.25 and |NES|>1. Based on the normalised enrichment score (NES), the enriched GOs and pathways with positive NES were governed by upregulated genes, and those with negative NES were governed by downregulated genes. The highest number of enriched GOs were found to be biological process GOs and the highest number of enriched pathways were found to be Reactome pathways. In breed-based analysis, the highest number of enriched GOs were identified in challenge 2-day data, while there were no enriched GOs in challenge 6-day data. In the case of pathways, the highest number of enriched pathways were found in challenge 2-day data and the least in non-challenged 10-day data. In challenge 2-day data, a greater number of the enriched biological process GOs were obtained under Cellular process level 2 GO, followed by the metabolic process. Several developmental, regulatory GOs were also observed, while very few enriched GOs were identified under response to stimulus GO. Most of the enriched pathways were found to be under Metabolism (KEGG), Human diseases (KEGG), Metabolism (Reactome) and Disease (Reactome) categories. A few enriched pathways were found under Environmental information processing (KEGG), Organismal systems (KEGG), Signal transduction (Reactome), Organelle biogenesis and maintenance (Reactome), Transcription (Reactome), Cell cycle (Reactome), Haemostasis (Reactome) and Immune system (Reactome) categories. In challenge 6-day data, although no enriched GOs were mentioned above, several enriched pathways were under the Disease (Reactome) and Signal transduction (Reactome) categories. A few enriched pathways were found under Metabolism (Reactome), Metabolism (KEGG), Environmental Information Processing (KEGG) and Human Diseases (KEGG) categories. In the 10-day challenge data, the highest enriched GOs were identified in Localization Level 2 GO, followed by Metabolic process, and only 1 GO in Cellular process GO. Most of the enriched pathways were found under the Disease (Reactome) category, and only 1 enriched pathway was found under each of the Signal Transduction (Reactome) and Human diseases (KEGG) categories. In non-challenged 2-day data, all the enriched GOs were under Cellular process GO. Most of the enriched pathways were found to be under the Metabolism (KEGG) and Disease (Reactome) categories. One enriched pathway was found under the Signal Transduction (Reactome), Organelle biogenesis and maintenance (Reactome), and Immune System (Reactome) categories. In non-challenged 6-day data, 2 enriched GOs were found under Metabolic process GO and 1 each under Cellular process and Localisation GO. Most of the enriched pathways were found under the Disease (Reactome) category, and few enriched pathways were found under Metabolism (Reactome), Transcription (Reactome), Organelle biogenesis and maintenance (Reactome), Haemostasis (Reactome), Metabolism (KEGG), Human diseases (KEGG) and Organismal systems (KEGG) categories. In non-challenged 10-day data, 2 enriched GOs under Cellular process GO and 1 each under response to stimulus, Metabolic process and Localization GOs were found. Only 1 enriched pathway was found in this data under the Metabolism (KEGG) category. In timepoint-based analysis, in Fayoumi, there were no enriched GOs in 2 vs. 6-day data, and 1 each enriched pathway was found under Human diseases (KEGG), Vesicle-mediated transport (Reactome), Cellular responses to stimuli (Reactome) and transport of small molecules (Reactome). In 2 vs. 10-day data, more enriched GOs were identified under Cellular process GO, followed by developmental process and biological regulation GOs. Several enriched pathways were found under the Disease (Reactome) category, and a few enriched pathways were also found under Metabolism of RNA (KEGG), Signal transduction (Reactome) and Cellular responses to stimuli (Reactome). There were no enriched GOs and pathways in 6 vs. 10-day data. However, in Leghorn, in 2 vs. 6-day data, the highest number of enriched GOs were obtained under cellular process and developmental process GOs. Several metabolic, developmental, and regulatory GOs were also observed; only 1 enriched GO was identified under the response to stimulus GO. The most enriched pathways were found under the Disease (Reactome) category. A few enriched pathways were also found under Metabolism (KEGG), Human Diseases (KEGG), Metabolism (Reactome), Cellular responses to stimuli (Reactome), Transcription (Reactome), Muscle contraction (Reactome), Chromatin organisation (Reactome), Developmental biology (Reactome) and Haemostasis (Reactome). In 2 vs. 10-day data, only 2 enriched GOs were obtained, one each under response to stimulus and metabolic process GOs. The most enriched pathways were found under the Disease (Reactome) category. A few enriched pathways were also found under Metabolism (KEGG), Immune system (Reactome), Vesicle-mediated transport (Reactome), Metabolism of proteins (Reactome) and Haemostasis (Reactome). Finally, in 6 vs. 10-day data, only 2 enriched GOs were obtained, one each under biological regulation and metabolic process GOs. However, there were no enriched pathways. The number of the total and enriched Gene Ontologies (BP, MF, CC) and Pathways (KEGG, Reactome) are shown in Table 3. Detailed information on enriched Gene ontologies was mentioned in Supplementary Table S6, and enriched pathways were mentioned in Supplementary Table S7.

**TABLE 3:** Table showing the number of Total and Enriched Gene Ontologies, i.e., Biological Process (BP), Molecular Function (MF) and Cellular Component (CC), Pathways, i.e., KEGG and Reactome identified in all the conditions in (A) Breed based and (B) Timepoint based analysis.

A		Non-Challenged			Challenged		
		2-day	6-day	10-day	2-day	6-day	10-day
BP	Total	2,314	1773	704	3,093	1,483	1,366
	Enriched	5	4	5	106	0	15
MF	Total	433	403	145	633	260	265
	Enriched	0	1	0	14	0	1
CC	Total	314	288	133	446	221	229
	Enriched	3	1	0	31	0	0
KEGG	Total	188	147	34	280	76	75
	Enriched	7	4	1	33	3	1
REACTOME	Total	470	311	87	863	170	185
	Enriched	16	14	0	23	14	12

B		Leghorn			Fayoumi		
		2 v/s 6	2 v/s 10	6 v/s 10	2 v/s 6	2 v/s 10	6 v/s 10
BP	Total	2,799	2026	347	758	1770	407
	Enriched	51	2	2	0	18	0
MF	Total	529	415	57	161	359	87
	Enriched	2	0	0	0	1	0
CC	Total	399	308	46	131	278	63
	Enriched	22	0	0	0	1	0
KEGG	Total	193	115	8	44	87	0
	Enriched	3	4	0	1	0	0
REACTOME	Total	547	347	15	57	382	35
	Enriched	22	16	0	3	14	0

### Co-expression analysis

In both breed-based and timepoint-based analysis, several co-expression modules were identified between DEGs and DElncRNAs. Supplementary Table S8 shows the statistics of the co-expression analysis of all the datasets. There were no outliers in all the datasets. Scale-free topology model, fit index cutoff, was set to 0.8, and soft power was chosen according to the index. In a few cases where the index was below the cutoff, a soft power of 9 was chosen, as mentioned in the user manual. The minimum module size was 15 for Breed-based analysis and 9 for timepoint-based analysis. In breed-based analysis, the highest number of modules were identified in challenged 2-day and 6-day samples. In timepoint-based analysis, most modules were identified in Leghorn 2 vs. 6-day samples.

### Functional analysis of lncRNAs

Most of the interactions were found to be trans-regulatory interactions, as shown in Table 4. About 93% were trans interactions, and 7% were found to be cis interactions. The lncRNAs were found to be downstream of the co-expressing gene in about 49.11% of cis interactions, upstream of the co-expressing gene in about 48.16% interactions, within the co-expressing gene in about 2.28% interactions, lncRNAs were spanning the 5′end in about 0.06% interactions and 3′end in about 0.38% interactions. The lncRNAs involved in cis interactions were functionally annotated from the functional annotation of the DEGs co-expressing with this lncRNA at all the different conditions. The results are mentioned in Supplementary Table S9. In breed-based analysis, challenge 2-day data showed the highest number of cis-interactions, DElncRNAs and DEGs, while non-challenged 10-day data showed the least number of cis-interactions, DElncRNAs and DEGs. In timepoint-based analysis, Leghorn 2 vs. 6-day data showed the highest number of cis-interactions, DElncRNAs and DEGs. In contrast, the Fayoumi 6 vs. 10 days data showed the least cis-interactions, DElncRNAs, and DEGs. There were no cis-interactions in Leghorn 6 vs. 10-day data. About 852 different lncRNAs were found to co-expressing with DEGs annotated with either an immune-related GO term or an immune system pathway, of which 435 lncRNAs were involved in more positive coregulations and 417 lncRNAs were involved in more negative coregulations.

**TABLE 4:** Table showing the number of cis and trans pairs of co-expressing genes and lncRNAs identified in (A) Breed-based and (B) Timepoint-based analysis.

A		Non-challenged			Challenged		
Timepoint		2-day	6-day	10-day	2-day	6-day	10-day
Total interactions		41,046	30,804	3,153	184,065	4,052	7,991
Trans		38,245	28,734	2,907	171,927	3,722	7,247
Cis	Total	2,801	2070	246	12,138	330	744
	Downstream	1,342	1,037	145	5,926	155	397
	Upstream	1,332	901	98	6,008	168	321
	Within gene	108	103	3	172	7	25
	At 3′ end	16	25	0	28	0	1
	At 5′ end	3	4	0	4	0	0

B		Leghorn			Fayoumi		
Timepoint		2v6	2v10	6v10	2v6	2v10	6v10
Total interactions		233,843	39,185	11	1,314	49,714	42
Trans		220,243	36,781	11	1,211	46,612	35
Cis	Total	13,600	2,404	0	103	3,102	7
	Downstream	6,969	1,112	0	49	1,485	0
	Upstream	6,414	1,177	0	52	1,617	3
	Within gene	181	87	0	2	0	4
	At 3′ end	28	23	0	0	0	0
	At 5′ end	8	5	0	0	0	0

### Gene-transcription factor interaction analysis

A total of 10 motifs were identified in the 5’ UTR of the DEGs expressed across all the conditions. Of these, 4 motifs (motifs 1, 2, 4 and 5) showed hits with transcription factors with a q-value of <0.05. About 47 transcription factors were identified for all the DEGs across all the conditions. Motif 1 showed hits with 21 transcription factors, motif 2 with 17 transcription factors, motif 4 with 30 transcription factors and motif 5 with 13 transcription factors. These transcription factors belong to 4 families: bHLH, E2F, ZBTB and zf-C2H2. The details of all the transcription factors identified using TomTom were mentioned in Supplementary Table S10A and details of transcription factors associated with immune-related genes were mentioned in Supplementary Table S10B.

### Gene -miRNA interaction analysis

A total of 661 miRNAs were found to interact with 431 DEGs across all six conditions. A total of 688 DEGs showed no miRNAs associated with them. About 79 miRNAs were found to target immune-related genes. The genes KIF13B and PRELP were targeted by the highest number of miRNAs, 50 miRNAs, and the miRNA gga-mir-1776 was found to target the highest number of DEGs, 47 DEGs. The details of microRNAs associated with DEGs were mentioned in Supplementary Table S10C.

### Interaction network analysis

**FIGURE 5:**
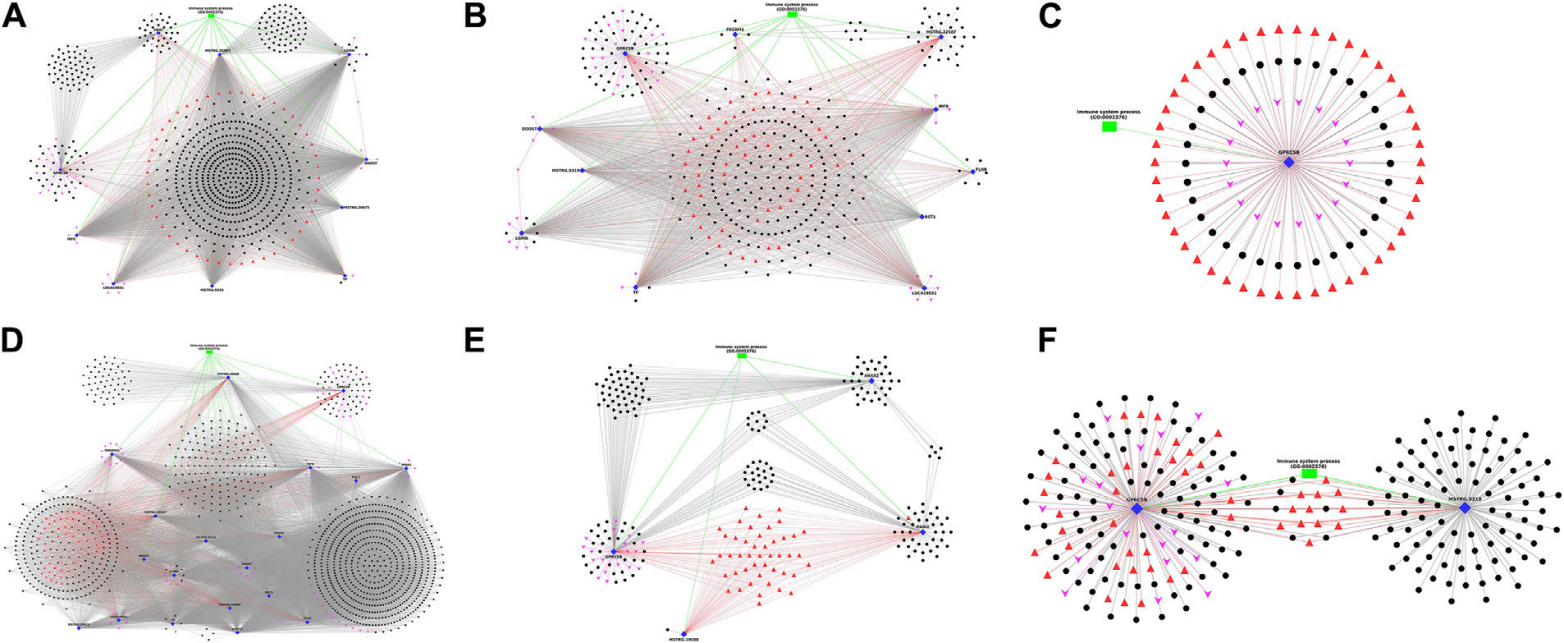
Figure showing the Network plot between immune annotated differentially expressed genes (Blue colour Diamond shaped), co-expressing differentially expressed lncRNAs (Black colour spherical shaped), associated transcription factors (Red colour triangular shaped), microRNAs (Pink colour arrow shaped) and biological process GOs (Green colour rectangular shaped) identified in breed based analysis. **(A)** non-challenged 2-day, **(B)** non-challenged 6-day, **(C)** non-challenged 10-day, **(D)** challenged 2-day, **(E)** challenged 6-day, **(F)** challenged 10-day.

The DEGs annotated with Immune-related Gene Ontology terms and Immune system pathways, along with co-expressing DElncRNAs, the associated transcription factors and miRNAs, were selected and plotted as network figures using Cytoscape (Shannon et al., 2003). In the network plot, Immune-annotated DEGs were represented by blue diamond nodes, co-expressing DElncRNAs with black spherical nodes, associated transcription factors with red triangular nodes, microRNAs with pink arrow nodes and biological process GOs with green rectangular nodes. The lncRNAs, TFs and microRNAs surrounding a specific gene were interacting with that particular gene and those in a separate cluster were interacting commonly with multiple genes. In breed-based analysis, six different network plots were generated and represented in Figure 5. In this figure, A represents network data obtained in non-challenged 2-day data. Of the 10 DEGs, the gene LGMN showed highest number of interactions with lncRNAs (557) and gene CD47 showed the least (96). The genes CD47, GPRC5B, LGMN and TF showed several lncRNAs which were co-expressing only with the respective gene. The lncRNAs and TFs in the central region denote the common one between all the DEGs. In Figure 5B represents network obtained from non-challenged 6-day data. Of the 11 DEGs, the gene DDOST showed highest interactions (179) and gene PECAM1 showed the least (11). The genes FLNB, GPRC5B, LGMN, MSTRG.22587, PECAM1 and TF showed several lncRNAs which were co-expressing only with the respective gene. In Figure 5C represents network obtained in non-challenged 10-day data. Only 1 gene GPRC5B was identified. In Figure 5D represents network obtained in challenged 2-day data. Of the 19 DEGs, the gene DDOST showed highest interactions (1,035) and gene FGL2 showed the least (51). The genes GPRC5B, LGMN and TF showed several lncRNAs which were co-expressing only with the respective gene. In Figure 5E represents network obtained in challenged 6-day data. Of the 4 DEGs, the gene GPRC5B showed highest interactions (104) and gene MSTRG. 19088 showed the least (1). All the 4 genes - ANXA2, GPRC5B, MSTRG.19088 and PADI2 showed several lncRNAs which were co-expressing only with the respective gene. In Figure 5F represents network obtained in challenged 10-day data. Of the 2 DEGs, MSTRG.9319 showed highest interactions (100) and gene GPRC5B showed the least (93). Both the genes showed several lncRNAs which were co-expressing only with the respective gene.

**FIGURE 6:**
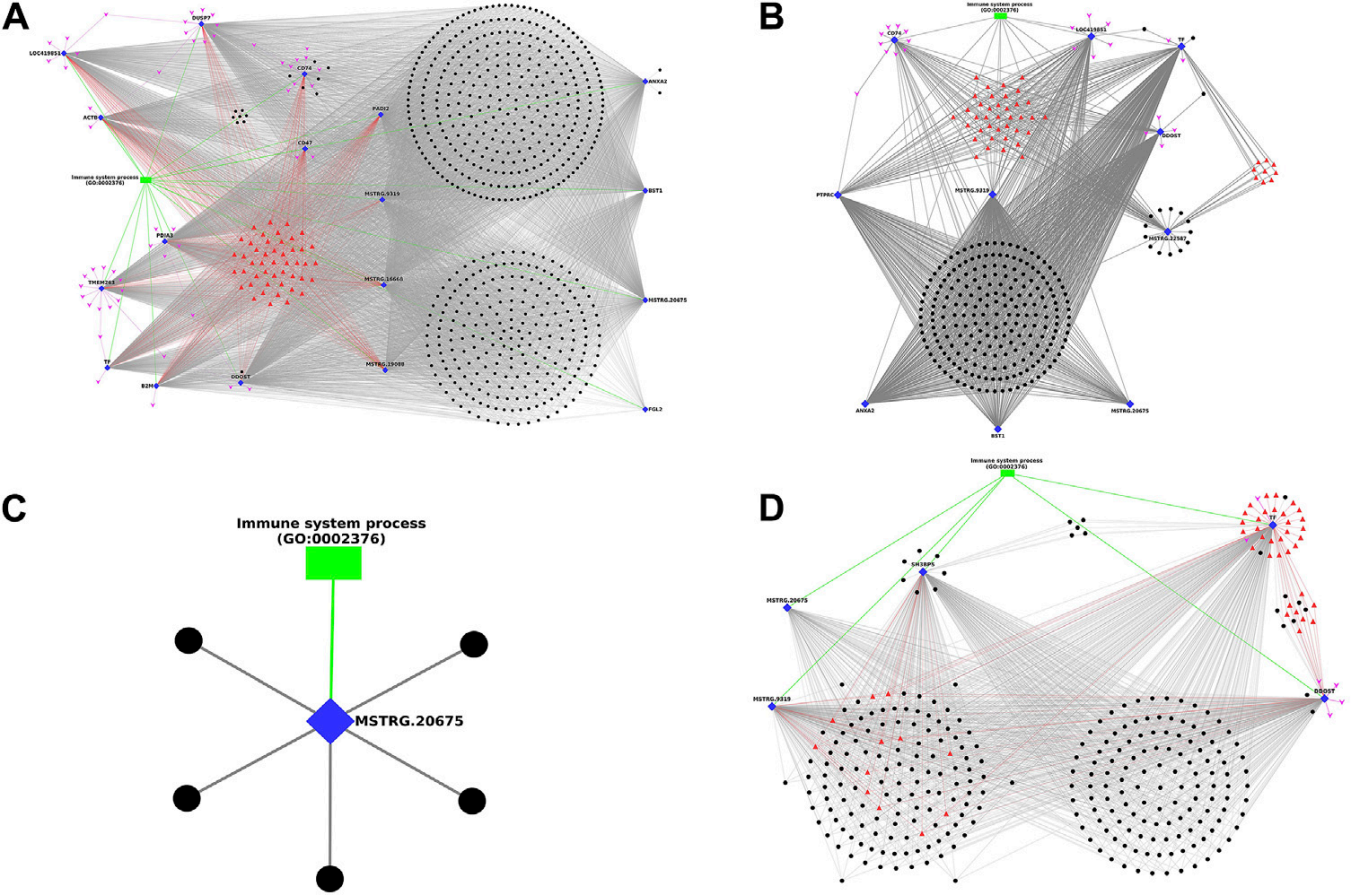
Figure showing the Network plot between immune annotated differentially expressed genes (Blue colour Diamond shaped), co-expressing differentially expressed lncRNAs (Black colour spherical shaped), associated transcription factors (Red colour triangular shaped), microRNAs (Pink colour arrow shaped) and biological process GOs (Green colour rectangular shaped) identified in timepoint based analysis. **(A)** Leghorn 2 v/s 6-day, **(B)** Leghorn 2 v/s 10-day, **(C)** Fayoumi 2 v/s 6-day, **(D)** Fayoumi 2 v/s 10-day.

In timepoint-based analysis, only four different network plots were generated and represented in Figure 6, as there were no interactions in Leghorn 6 v/s 10 DPC and Fayoumi 6 v/s 10 DPC data. In Figure 6A represents network obtained in Leghorn 2 v/s 6-day data. Of the 18 DEGs, the gene DDOST showed highest interactions (558) and genes CD47 and FGL2 showed the least (33). The genes ANXA2, CD74, DDOST showed several lncRNAs which were co-expressing only with the respective gene. In Figure 6B represents network obtained in Leghorn 2 v/s 10-day data. Of the 10 DEGs, the genes DDOST and TF showed highest interactions (214) and genes CD74 showed the least (11). The genes MSTRG. 22587 and TF showed several lncRNAs which were co-expressing only with the respective gene. In Figure 6C represents network obtained in Fayoumi 2 v/s 6-day. Only 1 gene MSTRG. 20675. In Figure 6D represents network obtained in Fayoumi 2 v/s 10-day. Of the 5 DEGs, the gene TF showed highest interactions (264) and the gene MSTRG. 20675 showed the least (113). The genes DDOST, SH3BP5 and TF showed several lncRNAs which were co-expressing only with the respective gene.

### QTL analysis

About 3,972 QTLs were obtained, including 729 Exterior, 1,006 Health, 654 Physiology and 1,583 Production QTLs. Of all six conditions, challenged 2-day samples showed the highest number of all types of QTLs (2032), and normal 10-day showed the lowest number of all QTLs (81). The details of the number of different types of QTLs obtained in each of the conditions are shown in Table 5.

**TABLE 5:** Table showing the number of differentially expressed genes associated with different types of QTLs obtained in (A) Breed-based analysis and (B) Timepoint based analysis.

A	Non-challenged			Challenged		
	2-day	6-day	10-day	2-day	6-day	10-day
Total Genes	320	225	31	855	85	132
Exterior QTL	133	101	17	367	43	68
Health QTL	201	135	23	506	55	86
Physiology QTL	121	97	10	336	35	55
Production QTL	302	214	31	820	84	127

B	Leghorn			Fayoumi		
	2v6	2v10	6v10	2v6	2v10	6v10
Total Genes	456	226	5	38	223	12
Exterior QTL	185	94	2	12	92	6
Health QTL	277	143	3	22	146	9
Physiology QTL	174	95	3	18	100	6
Production QTL	439	215	3	34	211	12

### Validation studies

**FIGURE 7:**
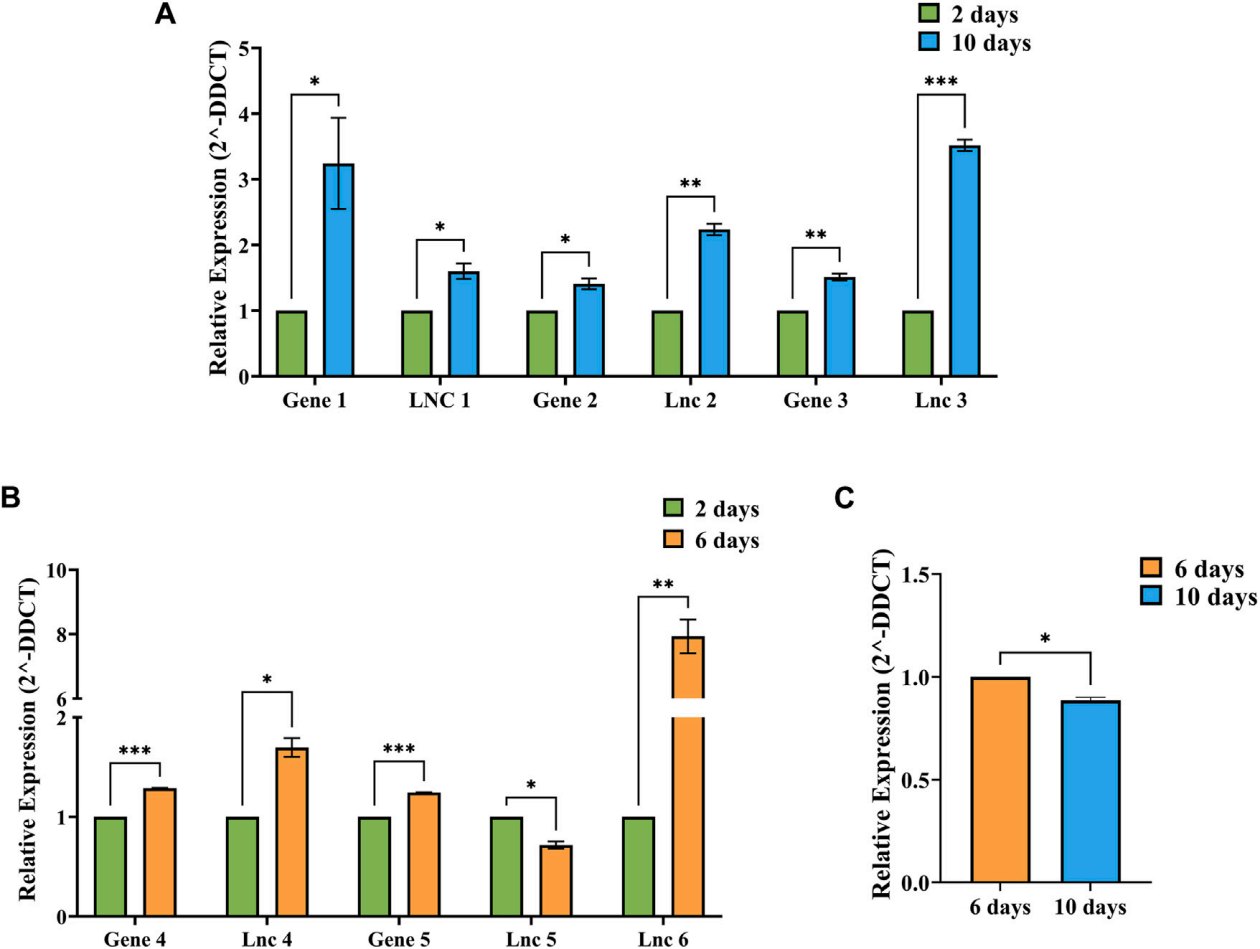
Figure showing the relative expression (2^-DDCT^) values of DElncRNAs and co-expressing genes identified in **(A)** 2 vs. 10-day, **(B)** 2 vs. 6-day and **(C)** 6 vs. 10-day conditions, with 2 days data represented as green bars, 6 days data as orange bars and 10 days data as blue bars. SEM values were plotted as error bars, and significance values (*p*) were represented by–*−*p*≤0.05, **−*p*≤0.01 and ***−*p*≤0.001.

The relative expression values between respective timepoints were calculated using the 2^-ΔΔCt^ method individually for each condition, i.e., 2 vs. 10-day, 2 vs. 6-day and 6 vs. 10-day Figure 7 shows the relative expression values of DElncRNAs and co-expressing DEGs identified in A: 2 vs. 10-day, B: 2 vs. 6-day and C: 6 vs. 10-day timepoints. The relative expression values determined through RT-PCR were consistent with the *in silico* analysis. In addition, the unpaired t-test results show that all the 7 lncRNAs and 5 genes showed significant differences.

## Discussion

Newcastle disease is a highly contagious disease that causes severe economic losses in the poultry industry worldwide. The harderian gland is one of the least studied tissues in chicken, and it is known to play a potential role in immune response. Our previous study identified the role of long noncoding RNAs and genes differentially expressing between normal and challenged chicken (challenge-based analysis). In this study, we have identified the role of long noncoding RNAs and genes that were differentially expressed between Leghorn and Fayoumi breeds of chicken (breed based analysis) and between the timepoints (2D, 6D, 10D) of challenged chicken (timepoint based analysis) of both the breeds separately.

In differential expression analysis, compared to challenge-based analysis, breed-based analysis and timepoint-based analysis showed more DEGs and DElncRNAs. A higher number of DEGs and DElncRNAs were found to be upregulated. This result shows higher differences between the two breeds than between the two disease conditions, i.e., challenge vs. non-challenge. Comparatively, the number of DElncRNAs is higher than the number of DEGs, but the expression levels of the DEGs were higher on average than that of DElncRNAs. In breed-based analysis, Challenge 2-day data showed more DEGs, followed by non-challenged 2-day data. The number of DEGs was found to be decreasing with time in both challenged and non-challenged animals. This analysis shows that both breeds showed a difference in gene expression in normal conditions and invoked a significantly different response against the NDV challenge.

In timepoint-based analysis, more DEGs and DElncRNAs were identified in the Leghorn datasets than in the Fayoumi datasets. At the 2 vs. 6-day condition, Leghorn showed a higher number of DEGs and DElncRNAs compared to Fayoumi, which shows that NDV challenge had showed a significant response right at the initial stages in susceptible Leghorn in comparison to resistant Fayoumi. At the 2 vs. 10-day condition and 6 vs. 10-day condition, the number of DEGs and DElncRNAs was similar in both Leghorn and Fayoumi.

Further, functional annotation has revealed that about 78 DEGs were annotated with immune-related GOs, i.e., Immune system process (GO:0002376), and about 240 DEGs were annotated with Immune system (Reactome) pathways. Commonly, 28 DEGs were found to be annotated with both immune-related GOs and pathways. About 290 DEGs were found to be annotated with either the Immune GO term, Immune system pathway, or both. In breed-based analysis, most immune-related genes were upregulated in Fayoumi in challenged and non-challenged groups. These DEGs were also highest at 2-day timepoints and decreased at 6-day and 10-day timepoints. There was a significant number of immune genes at the 2-day timepoint in non-challenged data, which indicates the differences between both breeds and indicates that these genes could play a potential role in Fayoumi being more resistant.

In contrast, the timepoint-based analysis shows that Leghorn shows more immune genes at 2 vs. 6-day and 2 vs. 10-day conditions, while Fayoumi shows more downregulated immune genes at 2 vs. 10-day and 2 vs. 6-day conditions. Several immune genes were upregulated in Leghorn at 2 vs. 6-day, and 2 vs. 10-day were downregulated in Fayoumi at 2 vs. 6-day and 2 vs. 10-day conditions. These results show that although Fayoumi shows a higher level of expression and more upregulated immune genes than Leghorn, it shows downregulation between two timepoints, which could mean that the higher expression had impacted the disease progress in Fayoumi in the initial stages. Later, the expression decreased due to a reduction in infection. As mentioned in previous studies, this result correlates with decreased NDV titer over time in Fayoumi.

The functional enrichment analysis observed that breed-based analysis challenge 2-day data showed the highest number of enriched GOs and pathways. In timepoint-based analysis, Leghorn 2 vs. 6-day data showed the highest number of enriched GOs and pathways. In breed-based analysis, challenged chicken showed a higher number of enriched GOs under cellular process and metabolic process GOs and few under response to stimulus GO, which were at the bottom of the gene list, i.e., downregulated at 2-day timepoint and at the top of the gene list, i.e., upregulated at 10-day timepoint. The non-challenged chicken also showed a similar type of enriched GOs with only cellular process GOs downregulated at the 2-day timepoint and cellular process GOs along with few metabolic process and response to stimulus GOs were upregulated at 6-day and 10-day timepoints. In timepoint-based analysis, Leghorn has shown several enriched GOs under cellular process, developmental process and metabolic process at the bottom of the gene list, i.e., downregulated at all three conditions–2 vs. 6-day, 2 vs. 10-day and 6 vs. 10-day. In the case of Fayoumi, enriched GOs were observed only in 2 vs. 10-day condition and under cellular process, biological regulation and developmental process GOs, which were at the top of the gene list, i.e., upregulated. This result shows that the NDV challenge has impacted and downregulated several non-immune Gene ontologies in Leghorn, while Fayoumi showed upregulation. These results show the clear differentiation in the expression of the genes between Leghorn and Fayoumi during NDV challenge and normal conditions. This finding also correlates with the transcriptomic expression patterns identified previously in the trachea transcriptome.

The co-expression analysis of DEGs and DElncRNAs showed that most of the gene-lncRNA interaction pairs were of trans type. As cis-lncRNAs were said to have a higher potential to regulate genes upstream or downstream, the cis-lncRNA-gene pairs were analysed further. It was observed that there were slightly higher numbers of positive and negative correlated interactions than negative. Almost 25% of the pairs involved the genes annotated with either an immune-related GO term or an immune system pathway. Positive and negative correlations were observed, with positive being slightly higher. In both challenge and timepoint-based analyses, Leghorn showed more cis-lncRNA interactions with immune-related genes than Fayoumi. While the breed-based analysis also showed several upregulated positive co-expressing lncRNAs. About 852 different DElncRNAs were found to be co-expressing with 178 different immune genes. About 12 different lncRNAs, i.e., TCONS_00110137, TCONS_00110138, TCONS_00116420, TCONS_00121555, TCONS_00126074, TCONS_00127291, TCONS_00128750, TCONS_00135305, TCONS_00137724, TCONS_00153297, TCONS_00138596 and TCONS_00151487 were found to be co-expressing with a set of same 24 immune genes, of which 21 genes were found to be having positive correlation with 10 DElncRNAs and negative correlation with 2 DElncRNAs.

In contrast, 3 DEGs were found to have a negative correlation with the same 10 DElncRNAs and a positive correlation with the other 2 DElncRNAs. The gene LYZ was found to have 145 different co-expressing DElncRNAs, with 68 positive and 77 negative interactions. Twelve different immune genes were found to be co-expressing with only 1 DElncRNA each, with 8 genes having a positive correlation and 4 genes having a negative correlation. Across all the conditions, except in challenge 2-day data, a higher number of gene lncRNA co-expression pairs were found to be positively co-expressing with immune-related genes than negative. In challenge 2-day data, more antagonistic co-expression pairs were observed. Compared to Fayoumi, a higher number of immune-related gene-lncRNA co-expression pairs were observed in Leghorn 2 vs. 6 days data, similar to genes. In addition, breed-wise QTL analysis showed that challenged 2-day samples showed the highest number of QTLs and non-challenged 10-day showed the lowest number of QTLs. The highest number of genes with Health QTL were also observed in challenge 2-day data. In timepoint-wise QTL analysis, Leghorn showed the highest number of genes with QTLS under the health QTL category compared to Fayoumi in all conditions.

Apart from this, several transcription factors and microRNAs interacting with the DEGs were also identified. Transcription factors interacting with the 28 immune annotated genes were identified, of which 5 DEGs showed no motifs with transcription factors, 2 DEGs showed all 4 motifs and all 47 transcription factors were found to interact with them. The remaining 21 DEGs showed 1-3 motifs and 12–46 transcription factors. Of the identified 47 transcription factors, 17 belong to zf-C2H2 (zinc finger Cys2–His2) family, which are known to play a vital role in disease and developmental process (Han et al., 2016), 14 belong to bHLH (basic helix-loop-helix) family, which play vital role in cell determination, differentiation and proliferation (Ledent and Vervoort, 2001), 1 belongs to ZBTB (Zinc finger and BTB domain containing) family, which play a vital role in differentiation, development and functioning of T-lymphocytes and related molecular mechanisms (Cheng et al., 2021), 1 belongs to E2F family, which regulate cell cycle and apoptotic processes (Xanthoulis and Tiniakos, 2013) and 14 were of unknown families. Along with the positive co-expressing lncRNAs, further studies can also use this transcription factor information to enhance the expression of the immune-related DEGs in the susceptible Leghorn breed to improve the overall resistance. Along with this, several microRNAs were identified to be associated with DEGs. In the case of the 28 immune-related genes, 13 genes showed no miRNAs. The remaining 15 genes were identified to be targeted by 79 different miRNAs. Of them, the miRNA gga-mir-2127 was identified to be targeting 3 genes–CD74, DUSP7 and PTPRC. Similarly, the miRNAs gga-mir-1647 (DUSP7 & GPRC5B), gga-mir-6556-5p (DDOST & LGMN), gga-mir-6561-5p (DUSP7 & IRF8), gga-mir-6587-3p (DUSP7 & LOC419851), gga-mir-6637-3p (ACTB & DUSP7), gga-mir-6649-3p (DDOST & TMEM263), gga-mir-6656-5p (DDOST & PDIA3), gga-mir-7456-5p (GPRC5B & TMEM263) and gga-mir-7460-5p (TF & TMEM263) were identified to be targeting 2 different genes each. Future research, including studies like small RNA-seq and chip-seq, can give further insights into miRNA and transcription factor expression levels during NDV challenge in chickens. This data, along with the information on transcription factors, microRNAs and co-expressing DElncRNAs identified in this study, can be used further to work on any specific genes that play a vital role in the immune system processes during NDV challenge in chickens to enhance their expression thereby improving the resistance in the susceptible breeds.

In our previous study on harderian gland data, it was observed that Leghorn showed a higher number of DEGs and DElncRNAs, especially those annotated with immune-related GO terms and pathways mainly in the comparison–Leghorn 6 DPC challenged vs. non-challenged. In comparison, resistant Fayoumi showed a lesser number of DEGs. The expression pattern of DElncRNAs was also found to be similar. In addition to this information, in the current study, Leghorn showed a higher number of upregulated immune-related genes than Fayoumi in timepoint-based analysis (between timepoints during the challenge), especially during the initial stages. This finding shows that Leghorn, being comparatively more susceptible to NDV than Fayoumi, showed several immune-related genes and positive co-expressing cis lncRNAs upregulated in challenged Leghorn when compared to non-challenged Leghorn and also in different timepoints during challenge. Surprisingly, Fayoumi, being comparatively resistant, showed little difference between challenged and non-challenged conditions and different time points of the challenge. This finding could also mean that Fayoumi might have similar immune gene and cis-lncRNA expression levels even during non-challenged conditions. The breed-based analysis, which compared Leghorn with Fayoumi in both challenged and non-challenged conditions separately, identified several immune-related genes and positive co-expressing cis lncRNAs to be upregulated in Fayoumi when compared to Leghorn in both challenged and non-challenged conditions. This result clearly shows that the differences in the expression of genes annotated with immune-related GO terms and pathways, i.e., immune-related genes and the co-expressing cis-lncRNAs between Leghorn and Fayoumi, and their role in the presence of differences in the resistance of Leghorn and Fayoumi chicken against NDV. This study is limited to *in silico* analysis of harderian gland tissue transcriptome data and experimental validation of expression of a few selected lncRNAs and co-expressing genes. The analysis of different tissues helps to understand the overall response of the host during disease. Further studies need to be done to unravel the mechanisms of co-expression and interaction of DEGs and DElncRNAs, which might be used for breed improvement.

## Conclusion

Newcastle disease is a highly contagious disease causing substantial economic impact. Leghorn and Fayoumi are the two breeds of chicken, with the first being comparatively more susceptible to NDV than the latter. In this study on harderian gland transcriptome analysis, we observed that the number of DEGs decreased with time in both challenged and non-challenged animals, and there was a significant difference in gene expression between Leghorn and Fayoumi, even in normal conditions. Several immune genes were differentially expressed at the 2-day timepoint in non-challenged data, which indicates the differences between both breeds, and this shows that these genes could play a potential role in Fayoumi being more resistant. In contrast, the timepoint-based analysis shows several genes annotated with immune-related GOs and pathways were upregulated in Leghorn and downregulated in Fayoumi between 2-day, 6-day and 10-day timepoints. These results show that, unlike Leghorn, Fayoumi shows a higher immune response initially and later, the response subsides with reduced infection. This result also correlates with decreased NDV titer over time in Fayoumi, as mentioned previously. In addition, the breed-based analysis showed several immune genes and positive cis-lncRNAs, which were upregulated in Fayoumi compared to Leghorn in both challenge and non-challenge conditions. Apart from immune-related genes, it was observed that there were several non-immune genes which were downregulated in Leghorn and upregulated in Fayoumi. A similar expression pattern was observed in the case of lncRNAs co-expressing with these genes. This study clearly shows the differences in the expression patterns of both genes and lncRNAs in Leghorn and Fayoumi during the NDV challenge in the harderian gland transcriptome. Further, in-depth analysis, including data from different tissues, will reveal several lncRNAs that might regulate the immune-related genes, making Fayoumi comparatively more resistant to NDV than Leghorn. The mechanism behind regulating the immune response and the role of the genes and lncRNAs can be utilised in breed-improvement programs to enhance the resistance of susceptible breeds.

## Data Availability

Publicly available datasets were analysed in this study. This data can be found here: EBI-ENA database with project ID PRJEB22672. The pipeline used in the study – FHSpipe can be found here: https://github.com/Venky2804/FHSpipe.

## Abbreviations

lncRNA, Long noncoding RNA; miRNA, Micro RNA; NDV, Newcastle Disease Virus; DEG(s), Differentially expressed gene(s); DElncRNA(s), Differentially expressed lncRNA(s); DPC, Days Post Challenge; EggNOG, Evolutionary Gene Genealogy Non-Supervised Orthologous Groups; KEGG, Kyoto Encyclopaedia of Genes and Genomes; WGCNA, Weighted Gene Correlation Network Analysis; MEME, Multiple Expectation Maximisations for Motif Elicitation; QTL, Quantitative Trait Locus.
