# Supplementary figures and images for: Breed and timepoint-based analysis of chicken harderian gland transcriptome during Newcastle disease virus challenge
Source: Front Mol Biosci. 2024 Jun 10;11:1365888. doi: 10.3389/fmolb.2024.1365888 (PMC11194529; doi:10.3389/fmolb.2024.1365888)

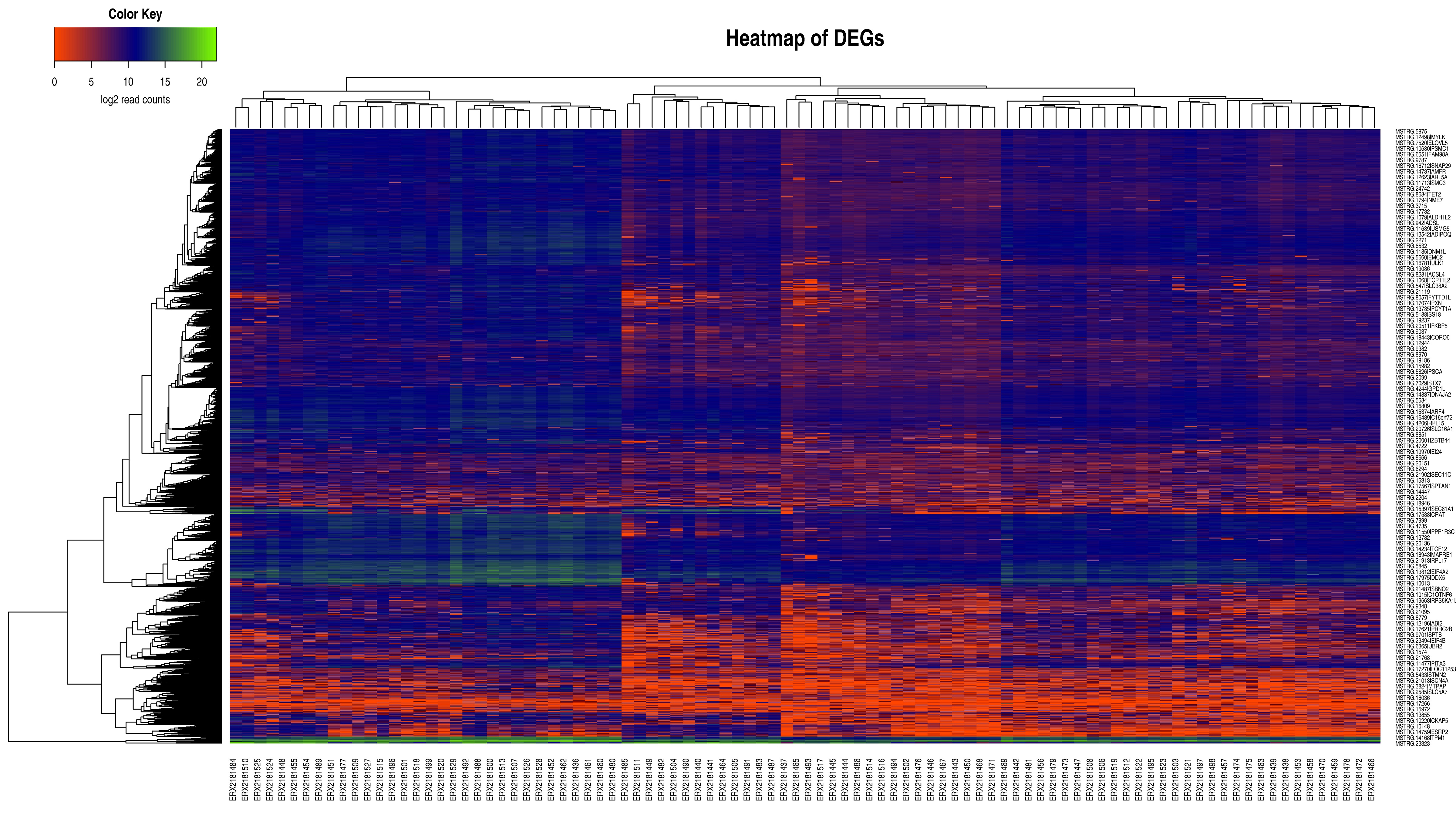

Supplement: Supplementary file 4 [file Image1.JPEG]

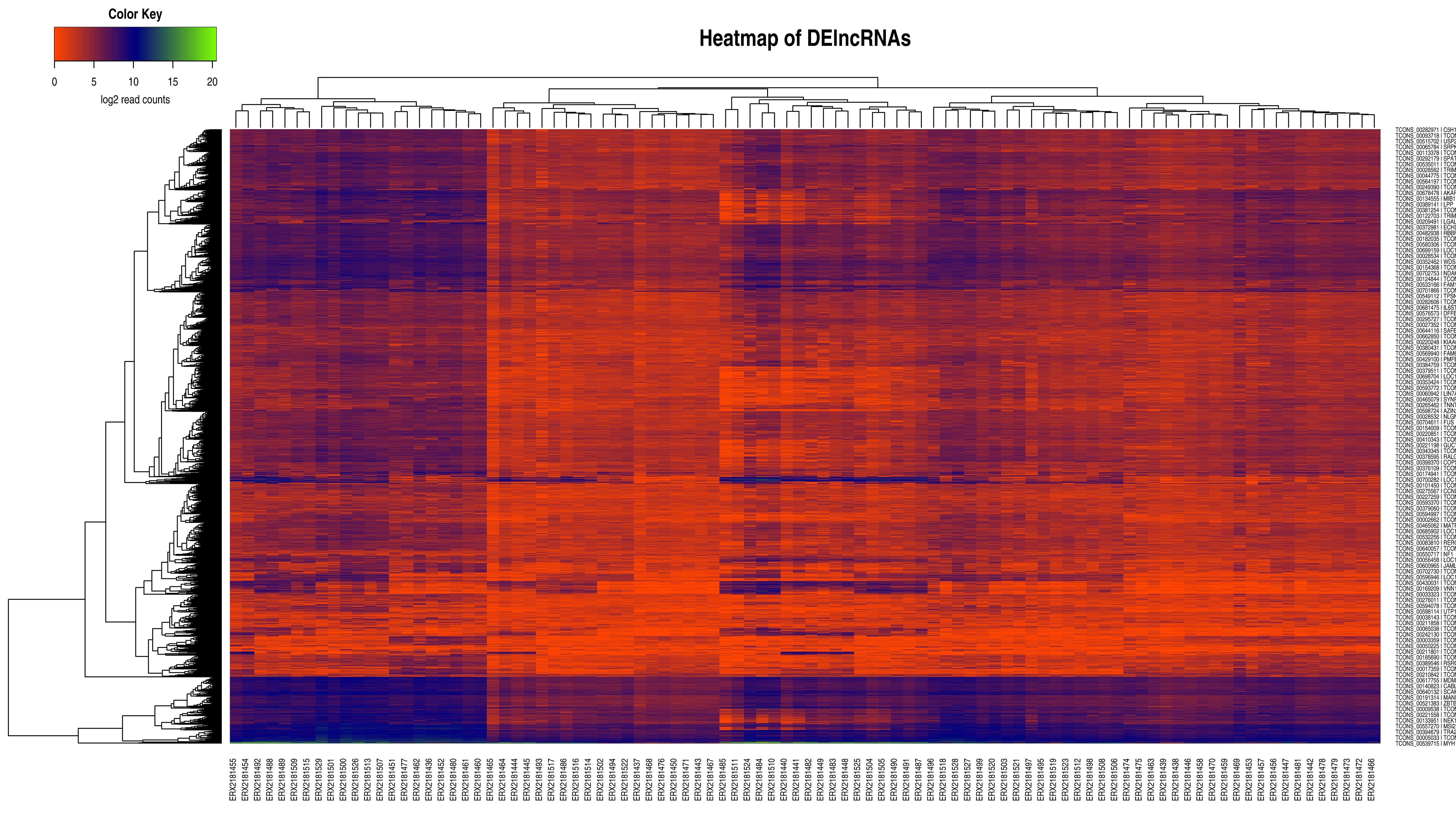

Supplement: Supplementary file 5 [file Image2.JPEG]
